# Supplementary material for: FAPbBr3 Perovskite Nanocrystals Embedded in Poly(L–lactic acid) Nanofibrous Membranes for Enhanced Air and Water Stability
Source: Membranes (Basel). 2023 Feb 26;13(3):279. doi: 10.3390/membranes13030279 (PMC10055916; doi:10.3390/membranes13030279)
Supplement: Supplementary file 1 [file membranes-13-00279-s001.zip › membranes-2177742-supplementary.pdf]

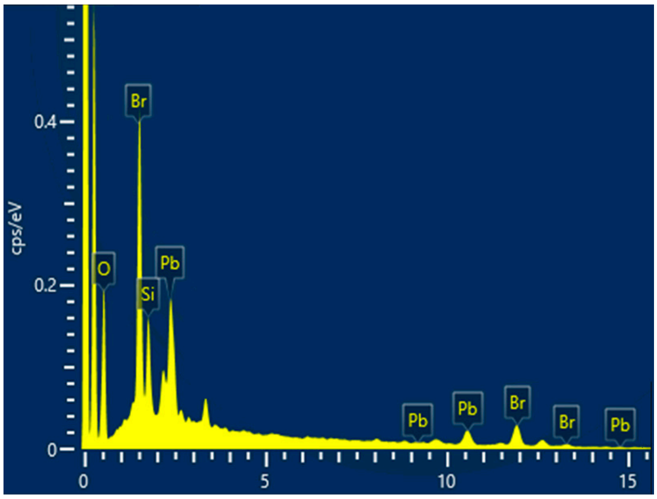

Figure S1: EDX analysis of $FAPbBr_3$ NCs-PLLA nanofibres

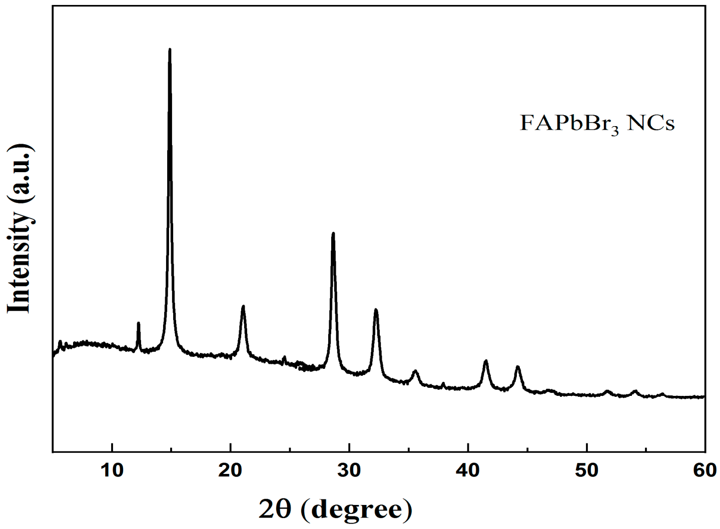

Figure S2: XRD pattern of $FAPbBr_3$ NCs
